# The V‐ATPase is expressed in the choroid plexus and mediates cAMP‐induced intracellular pH alterations

**DOI:** 10.14814/phy2.13072

**Published:** 2017-01-04

**Authors:** Henriette L. Christensen, Teodor G. Păunescu, Vladimir Matchkov, Dagne Barbuskaite, Dennis Brown, Helle H. Damkier, Jeppe Praetorius

**Affiliations:** ^1^ Department of Biomedicine, HealthAarhus UniversityAarhusDenmark; ^2^ Center for Systems BiologyProgram in Membrane Biology/Nephrology DivisionMassachusetts General Hospital and Harvard Medical SchoolBostonMassachusetts; ^3^ Department of Cellular and Molecular MedicineFaculty of Health and Medical SciencesUniversity of CopenhagenCopenhagenDenmark

**Keywords:** BCECF fluorometry, cerebrospinal fluid, fluorescence‐activated cell sorting, pH regulation

## Abstract

The cerebrospinal fluid (CSF) pH influences brain interstitial pH and, therefore, brain function. We hypothesized that the choroid plexus epithelium (CPE) expresses the vacuolar H^+^‐ATPase (V‐ATPase) as an acid extrusion mechanism in the luminal membrane to counteract detrimental elevations in CSF pH. The expression of mRNA corresponding to several V‐ATPase subunits was demonstrated by RT‐PCR analysis of CPE cells (CPECs) isolated by fluorescence‐activated cell sorting. Immunofluorescence and electron microscopy localized the V‐ATPase primarily in intracellular vesicles with only a minor fraction in the luminal microvillus area. The vesicles did not translocate to the luminal membrane in two in vivo models of hypocapnia‐induced alkalosis. The Na^+^‐independent intracellular pH (pH_i_) recovery from acidification was studied in freshly isolated clusters of CPECs. At extracellular pH (pH_o_) 7.4, the cells failed to display significant concanamycin A‐sensitive pH_i_ recovery (i.e., V‐ATPase activity). The recovery rate in the absence of Na^+^ amounted to <10% of the pH_i_ recovery rate observed in the presence of Na^+^. Recovery of pH_i_ was faster at pH_o_ 7.8 and was abolished at pH_o_ 7.0. The concanamycin A‐sensitive pH_i_ recovery was stimulated by cAMP at pH 7.4 in vitro, but intraventricular infusion of the membrane‐permeant cAMP analog 8‐CPT‐cAMP did not result in trafficking of the V‐ATPase. In conclusion, we find evidence for the expression of a minor fraction of V‐ATPase in the luminal membrane of CPECs. This fraction does not contribute to enhanced acid extrusion at high extracellular pH, but seems to be activated by cAMP in a trafficking‐independent manner.

## Introduction

Control of cerebrospinal fluid (CSF) pH is important, as it directly affects brain interstitial fluid (BIF) pH in the areas closest to the ventricle system (Okada et al. 1993). High BIF pH is associated with several forms of seizures, and is in some instances the leading cause of this condition (Schuchmann et al. 2006). The chemosensors of the brainstem may also be influenced by CSF acid–base status (Leusen 1954), which could have detrimental effects on the control of respiration. It seems counterintuitive that CSF has a low content of protein buffers, and pH is, therefore, believed to be buffered almost exclusively by the open CO_2_/HCO_3_^‐^ system (Lee et al. 1969). The fast equilibration of pCO_2_ between blood and CSF (Johnson et al. 1983) could lead to abrupt and large fluctuations in CSF pH. In dogs, however, CSF pH recovery during hypocapnia‐induced alkalosis is observed, which reaches significance after approximately 1 h and continues for the next 2–6 h (Kazemi et al. 1967). Neither the origin of the acid/base equivalents for CSF pH recovery, nor the responsible molecular mechanisms have been studied systematically. We have previously described NHE1 as an acid extruder in the luminal (i.e., CSF‐facing / apical) membrane of choroid plexus epithelium (CPECs) (Damkier et al. 2009), and it could, therefore, be a mechanism for lowering CSF pH. However, the low abundance of NHE1 in CPECs leads us to speculate that other acid extruders might co‐exist with NHE1 in the luminal membrane of CPE similar to what has previously been described for renal proximal tubule cells (Brown et al. 1988a). Such mechanisms might include other NHE isoforms, the vacuolar H^+^‐ATPase (V‐ATPase), the H^+^/K^+^‐ATPase, and the *Slc4a11* gene product.

The V‐ATPase is an obvious candidate for mediating regulated acid extrusion into the CSF. It is ubiquitously expressed in mammalian cells, where it acidifies several intracellular compartments, in particular the endosomal/lysosomal system (for review see (Nelson and Harvey 1999)). In specialized epithelial cells, such as kidney intercalated cells, the V‐ATPase is involved in maintaining whole body acid/base balance and responds to a systemic acidosis by increasing acid extrusion (Bastani et al. 1991; Sabolic et al. 1997). In the pigmented cells of ocular ciliary epithelium, V‐ATPase expression is mainly found in a subtype of cells and is thought to be involved in the formation of aqueous humor (Wax et al. 1997). Similarly, in the olfactory epithelium, V‐ATPase is expressed only in sustentacular and microvillar cells and is thought to regulate the pH of the mucus layer, thus being relevant in olfaction (Paunescu et al. 2008, 2012). In the inner ear, the V‐ATPase is important for maintaining high K^+^ balance of the endolymph to enable hearing (Karet et al. 1999). In the male reproductive tract, the V‐ATPase participates in maintaining an acidic luminal pH to mature and store spermatozoa (Brown et al. 1992), and in osteoclasts, the V‐ATPase in the ruffled membrane is vital for bone reabsorption (Baron et al. 1985).

The V‐ATPase is a multisubunit protein structurally similar to the mitochondrial F_1_F_0_ ATP synthases (for review see (Forgac 2007)). The V‐ATPases generate a transmembrane proton gradient using the energy provided by ATP hydrolysis, and consist of an integral membrane protein V0 domain and a cytoplasmic V1 domain joined by a stalk domain. The protein complex is composed of at least 14 different subunits, many of which exist in multiple isoforms; the V1 consists of subunits A‐H and the V0 consists of a, c, c'', d, e, and Ac45 (for review see (Nishi and Forgac 2002)). The B1 isoform is expressed in high abundance in the plasma membrane of kidney and epididymis proton secreting cells (Nelson et al. 1992; Breton et al. 1996). The B2 isoform is expressed in most cells in intracellular vesicles, such as lysosomes and endosomes and is usually not found in the plasma membrane (Puopolo et al. 1992). The regulation of acid secretion by the V‐ATPase in intercalated cells of the kidney collecting duct has been extensively studied. Trafficking of the V‐ATPase is regulated by increased luminal [HCO_3_^‐^], intracellular [HCO_3_^‐^], and the HCO_3_^‐^‐activated soluble adenylyl cyclase (sAC) with cAMP/PKA as downstream mediators (Paunescu et al. 2010; Gong et al. 2010). Thus, the current study was undertaken to explore the hypothesis that the V‐ATPase is expressed in the CPE and takes part in regulated acid extrusion into the luminal compartment, that is, the CSF.

## Materials and Methods

### Animals

In this study, we used, 8–12‐week‐old male C57BL/6 mice from Taconic (Denmark). All experiments were approved by the Danish Animal Experiments Inspectorate.

### FACS of CPE cells

Mice were anesthetised by isoflurane inhalation and euthanized by cervical dislocation. The choroid plexi (CP) from all brain ventricles were removed and placed in HEPES‐buffered saline (HBS) on ice (Table 1). Pooled CP tissues from 6 to 8 mice were incubated in 50 *μ*g/mL concanavalin A fluorescein (Vector Laboratories) in HBS for 10 min at 37°C, digested in 4 *μ*g/mL dispase (Invitrogen) and 4 *μ*g/mL collagenase B (Roche) in calcium‐free HBS for 30 min at 37°C, and incubated in a 1:1 mixture of TrypLE Select Enzyme (Thermo‐Fisher) and cell culture trypsin/EGTA (Thermo‐Fisher) supplemented with 1 mg/mL DNase (Sigma) for 10 min at 37°C. The preparation was passed through a 50‐*μ*m filter, and propidium iodide was added before Fluorescence‐activated cell sorting (FACS) for exclusion of dead cells. Cells were sorted into fluorescein‐positive and fluorescein‐negative samples by 4‐way purity sorting on a FACS Aria III (BD Biosciences). The cells were sorted using a 70‐*μ*m nozzle, at 70 psi and 12–20 kHz. After FACS, the samples as well as control CP samples were spun for 1 min in a table‐top micro‐centrifuge and the pellets were processed for total RNA isolation. Sample purity was validated by RT‐PCR with primers targeting the epithelial marker Claudin‐1 (*Cldn1*) and the endothelial marker Claudin‐5 (*Cldn5*).

**Table 1:** HEPES buffered solutions used for intracellular pH recording and Fluorescence‐activated cell sorting

Component	HBS	0Na^+^‐HBS	NH_4_Cl HBS	High‐K^+^ HBS	BBS
Na^+^	145.0	0.0	125.0	10.0	145.0
K^+^	3.6	3.6	3.6	138.6	3.6
Ca^2+^	1.8	1.8	1.8	1.8	1.8
Mg^2+^	0.8	0.8	0.8	0.8	0.8
NH_4_^+^	0.0	0.0	20.0	0.0	0.0
NMDG	0.0	145.0	0.0	0.0	0.0
Cl^−^	138.6	138.6	138.6	138.6	114.6
SO_4_^2−^	0.8	0.8	0.8	0.8	0.8
PO_4_^2−^	2.0	2.0	2.0	2.0	2.0
HCO_3_^−^	0.0	0.0	0.0	0.0	24.0
HEPES	10.0	10.0	10.0	10.0	10.0
Glucose	5.5	5.5	5.5	5.5	5.5
pH	7.4	7.4	7.4	7.4	7.4

### Reverse transcription ‐ polymerase chain reaction (RT‐PCR)

Mouse tissue was homogenized in TRI Reagent (Ambion), and total RNA was purified using the RiboPure^™^ Kit (Ambion). RNA samples were DNase treated (Invitrogen), and cDNA synthesis was carried out, using the SuperScript II Reverse Transcriptase system (Invitrogen). RT‐negative samples served as an internal control for the DNase treatment. PCR was performed on a DNA Engine DYAD Peltier Thermal Cycler (MJ Research) with initial denaturation at 95°C for 15 min, followed by 30–35 amplification cycles consisting of 30 sec of denaturation at 95°C, 30 sec of annealing at 58–60°C, and 30 sec of elongation at 72°C (see Table 2 for primer sequences and product sizes).

**Table 2:** Primers used for RT‐PCR in the study

Protein	Gene	Forward primer	Reverse primer	Base pairs
*β*‐actin	*Actb*	ACTGAGCTGCGTTTTACACCC	ACACAGAAGCAATGCTGTCACC	446
V‐ATPase A	*Atp6v1a*	CCAATCACCCCTTGCTTAC	TCTACTTTCCCATCAACCTCC	241
V‐ATPase B1	*Atp6v1b1*	AAACTACATCACCCACCCC	GCCAGAGCCATTGAAAATCC	298
V‐ATPase B2	*Atp6v1b2*	CAAACCCTACCTCTCAACTCC	ACGCAGAAACCGAAACCAC	466
V‐ATPase C1	*Atp6v1c1*	CCAAGCTGAACCACAATGAC	ATCCACGCAATAAACGCC	316
V‐ATPase C2	*Atp6v1c2*	AAGGGGAAAGCACACGAGAC	CTTGACTTGGGGACGATGAC	359
V‐ATPase a4	*Atp6v0a4*	CGACTACGATGACTCTTCCAAC	AATTCCACCCAATGCAGCC	552
V‐ATPase d1	*Atp6v0d1*	CGCTTTCATCATCACCATCAAC	ACAATCCCAGGACAATGCTAC	509
V‐ATPase d2	*Atp6v0d2*	AAAGCCAGCCTCCTAACTC	CTTGCAGAATTTGTAGAATGCC	525
Claudin 1	*Cldn1*	AGGTGCAGAAGATGTGGATGGC	AGAGGGAAGCAGCAGTTCACAG	527
Claudin 5	*Cldn5*	AATCAATTCCCAGCTCCCAGCC	TGAGTGCTACCCGTGCCTTAAC	443
Soluble AC 1	*Adcy10*	TGCATCTGAAATGTGCCCGC	TTTCAGCAGCCTTTCTCCCTCG	503
Soluble AC 1	*Adcy10*	TTTGCAGGTGATGCCTTGCTG	TCATGCTCCGATCACAGAGCTG	319

### Antibodies

An affinity‐purified V‐ATPase antibody raised in rabbit against the 70‐kDa A‐subunit of the protein (Hurtado‐Lorenzo et al. 2006) and a mouse monoclonal antibody raised against sAC (Zippin et al. 2003) were used in the study. Cy3‐conjugated donkey anti‐rabbit IgG (Jackson ImmunoResearch Laboratories) or donkey anti‐rabbit Alexa 488 antibodies (Invitrogen) and fluorescein isothiocyanate (FITC)‐conjugated donkey anti‐mouse IgG (Jackson ImmunoResearch Laboratories) were used as secondary antibodies.

### Immunoblotting

Mouse kidney was homogenized in ice‐cold dissection buffer (0.3 mol/L sucrose, 25 mol/L imidazole, 1 mmol/L ethylenediaminetetraacetic acid (EDTA), pH 7.2 containing 8.4 *μ*mol/L leupeptin (Calbiochem), 0.4 mmol/L Pefabloc [Roche]) and centrifuged at 4000 *g* for 15 min at 4°C. Sample buffer was added to the supernatant (final concentration: 0.1 mol/L sodium dodecyl sulfate and 0.04 mol/L dithiothreitol), pH 6.8. Dissected choroid plexus was dissolved directly in sample buffer and sonicated by 5 bursts 3 times at 60% power using a Model 150 V/T sonicator (BioLogics Inc.). The protein samples were heated at 65°C for 15 min and proteins were separated by 12.5% polyacrylamide gel electrophoresis and electro transferred onto a PVDF (Ambion) membrane. The membranes were blocked with 5% milk in PBS‐T (0.1 mol/L PBS (in mmol/L: 167 Na^+^, 2.8 H_2_PO_4_^‐^, 7.2 HPO_4_^2‐^, pH 7.4) with 0.1% vol/vol Tween), and incubated overnight at 5°C with primary antibody in PBS with 1% bovine serum albumin (BSA), and 2 mmol/L NaN_3_. After rinsing, the blots were incubated with horseradish peroxidase‐conjugated anti‐rabbit secondary antibody (Dako) and imaged (ImageQuant LAS4000, GE Healthcare).

### Tissue preparation and immunostaining

Mice were anesthetized by isoflurane inhalation and perfusion fixed with 4% paraformaldehyde (PFA) in PBS for 4 min via the left cardiac ventricle. Brains were removed and immersion post fixed in 4% PFA in PBS for 1 h at 4**°**C. Tissues were rinsed in PBS and dehydrated in increasing concentrations of ethanol (70%, 96% and 99%, 2 h in each) before they were placed in xylene overnight. Tissues were infiltrated with paraffin for 1 h at 60°C. 2‐*μ*m sections were cut on a microtome (RM 2165; Leica Microsystems).

Tissue slides were deparaffinized in xylene, rehydrated in decreasing concentrations of ethanol (99%, 96%, and 70%), and rinsed in PBS. For antigen retrieval, slides were treated with 1% (w/v) SDS in PBS for 4 min and washed in PBS. Sections were blocked with 1% (w/v) bovine serum albumin in PBS containing 0.02% sodium azide for 20 min and incubated with primary antibody for 90 min at room temperature. Slides were washed in PBS, incubated with secondary antibody for 60 min at room temperature, washed again and mounted with coverslips using Vectashield mounting medium (Vector Laboratories Inc.) containing DAPI, or incubated with Topro3 (Invitrogen) nuclear stain before mounting with glycergel anti‐fade (Dako). An 80i epifluorescence microscope (Nikon Instruments) equipped with an Orca 100 CCD camera (Hamamatsu) or a DM IRE2 inverted confocal microscope (Leica Microsystems) was used to acquire the digital images.

### Analysis of cellular V‐ATPase distribution

Quantification of V‐ATPase distribution was performed by line scanning (ImageJ software, National Institutes of Health) across cells with a visible nucleus. The line was perpendicular to the basement membrane and expanded to a band with a width of 21 pixels and placed in the mid‐nuclear plane of each cell containing a nucleus in all images (2–8 images per mouse). The number of bins (measurement points) was compressed to 50 (or 40, in Fig. 5) for each cell and the average staining intensity for each bin was calculated for each of the ten mice, where bin #1 represents the basal end of the cells. At the luminal end, the last 5 bins most likely represent the brush border. To correct for background staining, the lowest staining intensity from each mouse was subtracted from each of the other bins from the same mouse. After this, the average staining intensity for each of the 50 (or 40) bins was calculated for all nucleated cells for each mouse and averaged for every group of 5 mice (hyperthermia‐treated mice and control mice).

### Immunogold electron microscopy

The choroid plexus that was fixed with 4% paraformaldehyde in PBS (0.9% NaCl in 10 mmol/L phosphate buffer, pH 7.4) was dehydrated through a graded ethanol series to 100% ethanol and subsequently infiltrated, embedded, and polymerized in LR White resin (EMS) at 50°C as previously described (Paunescu et al. 2010). Thin choroid plexus sections were cut to approximately 70‐nm sections on an EM UC7 ultra‐microtome (Leica Microsystems) and collected onto formvar‐coated grids. The grids were floated on drops of primary rabbit anti‐V‐ATPase A subunit antibody diluted 1:50 in antibody diluent (Dako) for 2 h at room temperature, rinsed on drops of PBS, and floated on drops of goat anti‐rabbit IgG antibody coupled to 15‐nm gold particles diluted 1:15 in antibody diluent for 1 h at room temperature. The grids were rinsed, poststained on drops of 2% uranyl acetate for 5 min, rinsed, and dried. The choroid plexus sections were examined in a JEM‐1011 transmission electron microscope (JEOL) at 80 kV. Images were acquired using an AMT XR60 digital imaging system (Advanced Microscopy Techniques).

### Intracellular pH (pH_i_) measurements

Experiments were conducted as described previously (Damkier et al. 2009). Isolated CP tissues were digested into single‐layered cell clusters by 4 *μ*g/mL dispase (Invitrogen) and 4 *μ*g/mL collagenase B (Roche) in calcium‐free HBS (Table 1) at 37°C for 30 min. The digested cell clusters were mounted on Cell‐Tak‐coated (BD Biosciences) coverslips for 10–15 min at 37°C and loaded for 10 min with the pH‐sensitive probe BCECF‐AM (2 *μ*mol/L, Invitrogen). Coverslips were mounted in a closed perfusion chamber (RC‐21BR; Harvard Apparatus) and placed on an inverted microscope stage inside a 37°C dark box. Cells were allowed to equilibrate to a baseline level in HBS before cellular acidification by an NH_4_Cl prepulse for 3–5 min (NH_4_Cl HBS, Table 1) followed by Na^+^‐free HBS (0Na^+^ HBS, Table 1). Each experiment was concluded with a one‐point calibration in pH 7.0, high K^+^ HBS (Table 1) containing 10 *μ*mol/L nigericin.

Till Vision software (Till Photonics) was used to control monochromator wavelength alternating between 490 nm and 440 nm, exposure time (20 msec), frequency (1 Hz), and binning (to 512 × 512 pixel images). The light emission at 510–535 nm was recorded by a 12‐bit cooled monochrome CCD camera (QImaging, Retiga EXi) and data were collected from user‐defined regions of interest (ROIs) of individual cells after background subtraction. The fluorescence ratio (490/440 nm) was calibrated to pH by clamping pH_i_ stepwise from pH 8–6 in high‐K^+^ HBS with 10 *μ*mol/L nigericin (Boyarsky et al. 1988). The rate of pH_i_ recovery (dpH_i_/dt) was determined as the pH_i_ change in 30 sec after peak minimum pH_i_.

### Measurement of membrane potential (*V*_m_) in isolated CPE cells

Isolated clusters of CPECs were plated in Cell‐Tak (BD Biosciences) coated culture dishes (Falcon, Becton Dickson) containing HBS. Isolated cells and small pieces of epithelia were allowed to adhere to the chamber bottom for 15 min prior to Vm recording (Kotera and Brown 1994). All recordings were made at room temperature with borosilicate glass patch pipettes (Harvard Apparatus) with resistances in the range of 2–5 MΩ. Recordings were made with an Axopatch 200B amplifier (Axon Instruments Inc., CA) by switching from the voltage‐clamp to current‐clamp mode. First, a whole‐cell configuration was established in voltage‐clamp mode (seal resistance >1 GΩ; access resistance 5–10 MΩ) and then immediately switched to the current‐clamp mode to detect Vm. Vm detected immediately after establishing the whole‐cell configuration (within no more than 1 min) was recorded for further analyses. Pipette solution in these experiments contained (in mM): 140 K^+^, 4 Na^+^, 2 Mg^2+^, 120 aspartate, 24 Cl^‐^, 2 ATP^2‐^, 0.5 EGTA, 5 HEPES and adjusted to pH = 7.4, while HBS was used as bath solution.

### Respiratory alkalosis (hypocapnia) by hyperthermia and hypoxia

For hyperthermia, a heating lamp was used to increase the temperature of a container to 42°C. The mice were placed in the container individually for 5–7 min, thereby inducing hyperventilation and causing wash out of CO_2_ before isoflurane anesthesia was induced. Controls were placed in the chamber for the same amount of time but at room temperature. For hypoxia, the mice were placed inside a chamber containing a gas mixture of 12% O_2_ in 88% N_2_ for 5 min before induction of isoflurane anesthesia in the same gas mixture. The control mice inhaled normal air. For both experiments, blood samples were drawn from the right atrium of the heart and the mice were perfusion fixed with 4% PFA in PBS via the left ventricle. Blood was analyzed on an ABL80 Flex blood gas analyzer (Radiometer).

### Stereotactic injection of cAMP into brain ventricles of mice

Mice were anesthetized by a bolus intraperitoneal injection of ketamine (100 mg/kg) and xylazine (10 mg/kg) followed by continuous injections of smaller doses of anesthesia as needed. Each mouse was placed in stereotactic equipment (David Kopf Instruments) and a Hamilton syringe was inserted 2.5 mm through the skull into the lateral ventricles at coordinates 0.8 mm lateral, and 0.1 mm caudal to bregma. A volume of 5.5 *μ*L 10 mmol/L 8‐CPT‐cAMP (Sigma‐Aldrich) or vehicle (artificial CSF/aCSF, Alzet) was slowly injected (0.5 *μ*L/min). After 30 min, the mouse was perfusion fixed and the brain was subjected to immunohistochemistry.

### Statistics

Mean values for pH_i_ recovery rate were compared by nonparametric ANOVA with Dunnet posttest, as the data did not pass the normality test. V‐ATPase‐staining intensities and blood gas values were analyzed by a two‐tailed t‐test. For all analyses, *P *< 0.05 was considered significant.

## Results

### The V‐ATPase is expressed mainly in intracellular vesicles of CPECs

**Figure 1:**
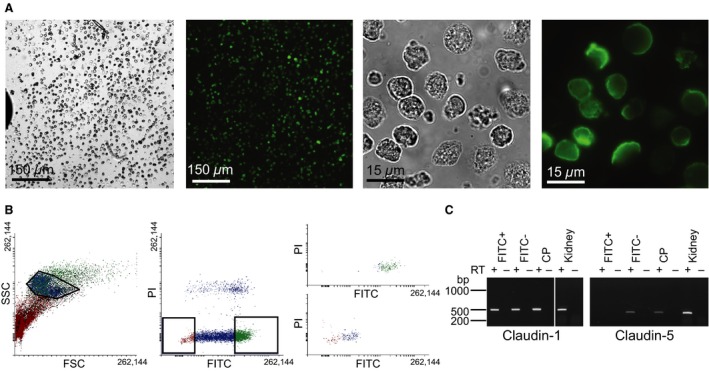
FACS isolation of mouse CPECs. Mouse choroid plexi were surface‐labeled with fluorescent concanavalin A, and enzymatically digested into cell suspensions. (A) The left panels show transmitted light and fluorescence micrographs of a cell suspension at low magnification, while the right panels show high magnification micrographs of the same cells. (B) The left panel shows the data from FACS isolating CPECs based on side and forward scatter (SSC and FSC, respectively). The frame represents selected cells for further gating. The middle panel shows selection of green fluorescence (FITC) after detection of singlets, while excluding damaged cells based on propidium iodide uptake (PI), as indicated by the boxes. The right panels illustrate a recount of the isolated cell suspension, where the top panel represents analysis of fluorescein positive cells and the bottom panel analyses the fluorescein negative cell population. (C) The cDNA samples from cells isolated by FACS were subjected to PCR with primers targeting epithelial marker claudin‐1 and endothelial marker claudin‐5. RT + and – indicates the presence or omission of reverse transcription, respectively. FITC‐ denotes unlabeled CP cells, FITC+ represents isolated fluorescein‐lectin labeled cells, CP is the entire choroid plexus, and whole kidney cDNA was used as positive control. CPECs, choroid plexus epithelial cells; FACS, Fluorescence‐activated cell sorting.

**Figure 2:**
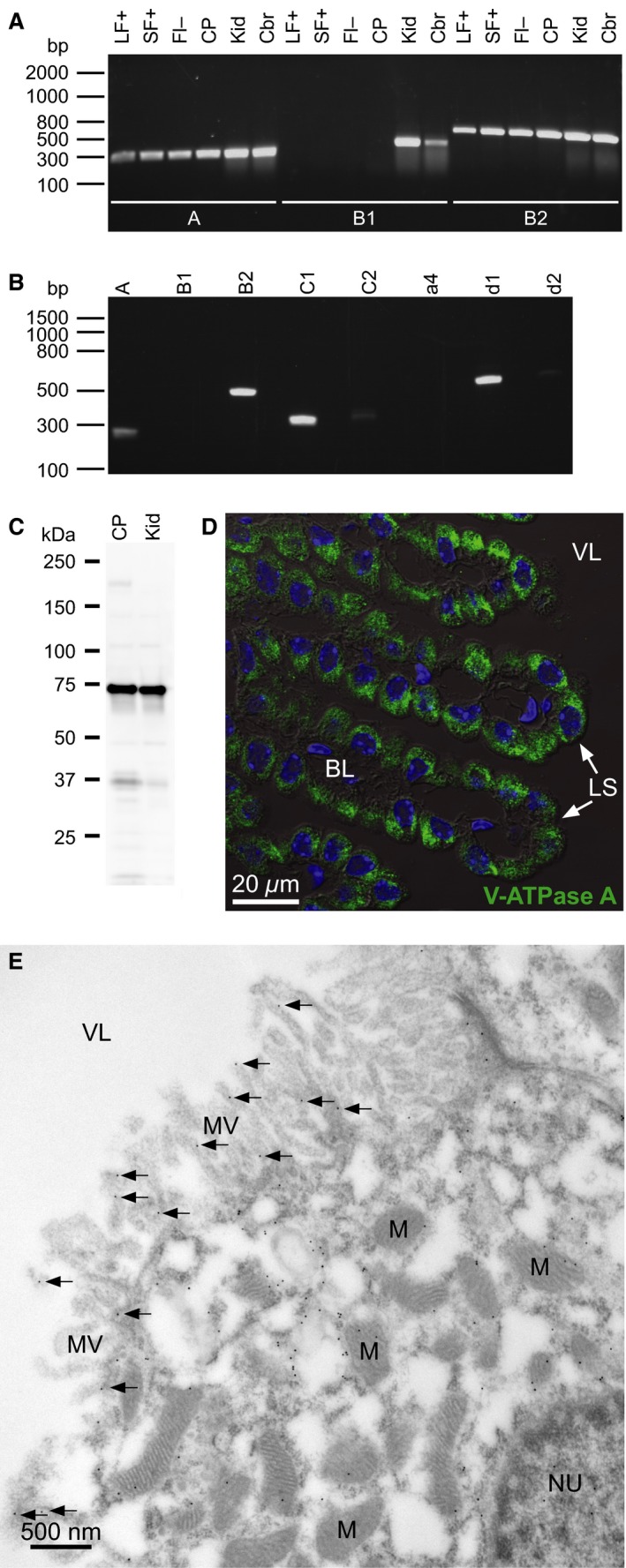
The V‐ATPase is expressed in the mouse CPECs and localizes mainly to intracellular vesicles. (A) RT‐PCR analysis of V‐ATPase subunit expression. FACS‐sorted mouse CPEC samples were reverse transcribed, and the resulting cDNA samples were subjected to PCR with primers targeting V‐ATPase subunits A (*Atp6v1a*), B1 (*Atp6v1b1*), and B2 (*Atp6v1b2*). LF+ indicates large fluorescein positive cells, SF+ represents small fluorescein positive cells, FI‐ specifies fluorescein negative cells, CP denotes entire choroid plexus, while Kid and Cbr are abbreviations for kidney and cerebral control samples. (B) PCR analysis of whole CP cDNA samples with primers targeting V‐ATPase subunits A, B1, B2, C1 (*Atp6v1c1*), C2 (*Atp6v1c2*), a4 (*Atp6v0a4*), d1 (*Atp6v0d1*), and d2 (*Atp6v0d2*). (C) Immunoblot analysis of V‐ATPase A subunit expression in CP. Proteins were extracted from the entire isolated CP and subjected to SDS‐PAGE and immunoblotting. A kidney protein sample (Kid) served as positive control. (D) Immunolocalization of V‐ATPase A subunit in the CPECs. Semi‐thin sections of mouse brain were subjected to immunofluorescence analysis. Green color indicates V‐ATPase A staining, blue color represents DNA counter‐staining in nuclei. (E) Electron micrograph of choroid plexus from a control mouse stained with an antibody against the A subunit of the V‐ATPase. 15 nm gold particles show the localization of this subunit in the cell. Most of the gold particles are over intracellular structures, with only very sparse labeling of the apical microvilli. NU, nucleus; M, mitochondria; MV, microvilli, VL, ventricle lumen; CPE, choroid plexus epithelium; FACS, Fluorescence‐activated cell sorting; CPEC, choroid plexus epithelial cell; LS, luminal (CSF‐facing) surface; BL, basolateral interstitium.

Luminal membrane labeling with fluorescent lectin, cell separation (Fig. 1A), and FACS yielded 99.8–100% pure fluorescein‐positive CPECs (Fig. 1B). RT‐PCR on cDNA from FACS isolated CPECs for the epithelial marker claudin‐1 and the endothelial marker claudin‐5 (Fig. 1B), as well as collagen I (fibroblastic marker, not shown) validated that these samples were pure suspensions of CPE cells. RT‐PCR demonstrated expression of V‐ATPase subunits A and B2, but not B1, in the CPECs (Fig. 2A). Additionally, we detected C1, C2, and d1 subunits in nonsorted CPEC samples (Fig. 2B). Immunoblotting produced a V‐ATPase A‐immunoreactive band of a size corresponding to the calculated MW of 68 kDa in both CP and kidney samples (Fig. 2C). Immunohistochemical analysis localized the V‐ATPase A subunit mainly to intracellular compartments in the CPECs (Fig. 2D). A similar staining pattern was seen using a B2 subunit‐specific antibody (not shown). Immunogold electron microscopy supported the predominant intracellular vesicular localization (Fig. 2E). Sparse labeling was observed in the microvilli of the CPECs. Thus, multiple subunits of the V‐ATPase are expressed in CPECs and mainly observed in intracellular vesicles.

### Na^+^‐independent pH_i_ recovery in CPECs depends on extracellular pH (pH_o_) but is insensitive to concanamycin A

**Figure 3:**
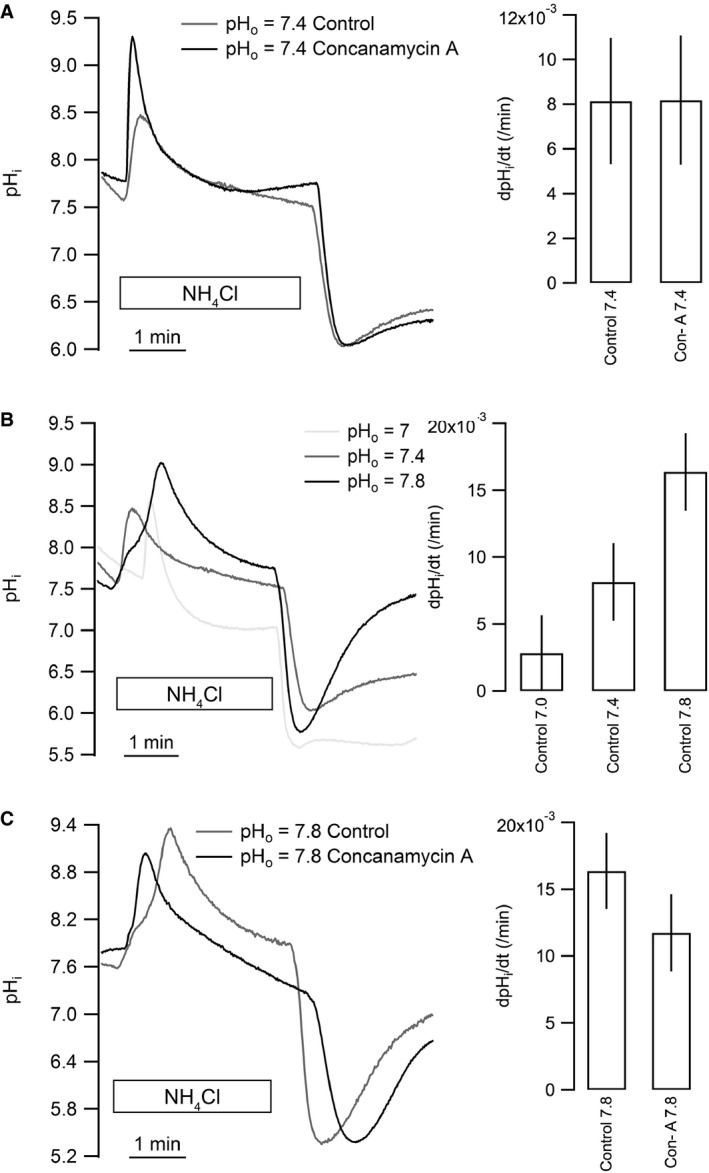
Na^+^‐ and CO_2_/HCO_3_^‐^‐independent acid extrusion does not involve V‐ATPase activity in isolated CPECs. Isolated clusters of CPECs were loaded with BCECF and pH_i_ was monitored during NH_4_Cl prepulse acid‐loading and pH_i_ recovery. (A) Effect of the V‐ATPase inhibitor concanamycin A on pH_i_ recovery at pH_o_ 7.4 (*n* = 8 for control and *n* = 7 for 100 nmol/L concanamycin A). (B) Following acidification, the extracellular pH (pH_o_) was clamped to 7.0, 7.4, or 7.8 as indicated (left). The bar graph (right) shows mean values ± SEM for net acid extrusion rate in CPECs (for pH_o_ 7.0, 7.4, and 7.8: *n* = 4, 8, and 6, respectively). (C) Similar recordings and summarized results showing the effect of concanamycin A (100 nmol/L) on pH_i_ recovery at pH_o_ 7.8 (*n* = 6). CPECs, choroid plexus epithelial cells.

In order to assess the acid‐extruding potential of the V‐ATPase in CPE cells, isolated choroid plexus cells were acidified by an NH_4_^+^ prepulse and the Na^+^‐independent pH_i_ recovery was estimated from the point of maximal acidification. Concanamycin A was applied to determine the contribution of V‐ATPase activity to the Na^+^‐independent pH_i_ recovery. No significant effect of concanamycin A on pH_i_ recovery was found compared to control conditions at pH_o_ 7.4 (n.s., *n* = 7 and 8 respectively, Fig. 3A). The initial pH_i_ following acidification was 5.97 ± 0.14 in the control experiments and 5.94 ± 0.18 in the concanamycin A experiments. Figure 3B shows that pH_i_ recovery after acidification was almost absent at pH_o_ 7.0, while it was more prominent at pH_o_ 7.4 and even more so at pH_o_ 7.8. The dpH_i_/dt was not significantly different from zero at pH_o_ 7.0 (n.s., *n* = 4), while dpH_i_/dt increased with increasing pH_o_ (*P* = 0.0063 between pH_o_ 7.0 and 7.8, *n* = 4 and 7, respectively). The initial pH_i_ among the three pH_o_ levels was 6.29 ± 0.31 at pH_o_ 7.0, 5.97 ± 0.14 at pH_o_ 7.4, and 6.34 ± 0.27 at pH_o_ 7.8 (n.s.). As illustrated in Figure 3C, concanamycin A seemed to induce a reduction of the pH_i_ recovery at pH_o_ 7.8, although the change was not statistically significant compared to control (n.s., *n* = 6 and 7, respectively). The initial pH_i_ was 6.34 ± 0.27 in the control experiments and 6.32 ± 0.19 in the concanamycin A experiments (n.s.). Thus, the V‐ATPase does not seem to contribute significantly to pH_i_ recovery in acidified CPE cells.

### Membrane potential (*V*_m_) of isolated murine CPECs and equilibrium potentials for H^+^

To allow computation of whether passive H^+^ flux might mediate the pH_i_ recovery, we estimated the Vm of isolated CPE cells. Clusters of CPE cells were isolated, and the *V*_m_ was determined by whole cell patch clamping. In 14 patches from 4 mice, we determined a *V*_m_ of −47.4 ± 2.1 mV. Based on the measured pH_i_ and the given pH_o_ values, the equilibrium potential for H^+^, Veq(H^+^), is −42 mV at pH_o_ 7.0, −67 mV at pH_o_ 7.4, and −92 mV at pH_o_ 7.8.

### The Na^+^‐independent pH_i_ recovery is modest compared to the NHE activity in CPECs

**Figure 4:**
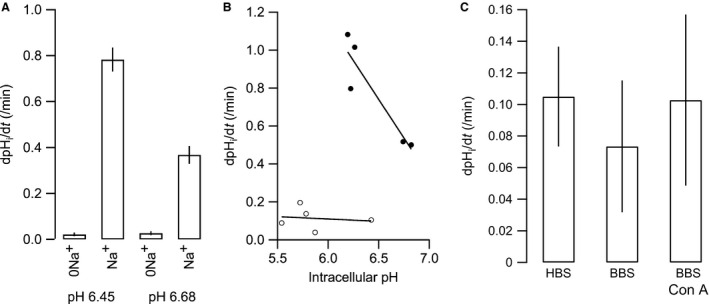
Na^+^‐independent acid extrusion in isolated CPECs is minute compared to NHE activity at low pH_i_. (A) The calculated pH_i_ recovery rates after acidification in the absence and presence of 145 mmol/L Na^+^ at pH_i_ 6.45 (0Na^+^ and Na^+^, respectively, *n* = 4), and at pH_i_ 6.68 (0Na^+^ and Na^+^, respectively, *n* = 6). (B) The recovery rate as a function of pH_i_ in the absence (open circles) and presence of 145 mmol/L Na^+^ (closed circles). (C) Comparison of the Na^+^‐independent pH_i_ recovery in the absence (HBS) and presence of CO_2_/HCO_3_^‐^, with and without 100 nM concanamycin A (BBS+Con A and BBS, respectively, *n* = 3).

To compare the relative contribution of NHE activity (Na^+^‐dependent recovery) and V‐ATPase/leak‐mediated H^+^ translocation (Na^+^‐independent recovery), the rate of pH_i_ recovery was compared directly before and after the addition of Na^+^ in acidified cells in the same experiment. The dpH_i_/dt in Na^+^‐free HBS amounted to 2.63 ± 1.02% of dpH_i_/dt in Na^+^ containing HBS at pH_i_ 6.45 ± 0.14 (*P *< 0.0001, *n* = 6, Fig. 4A and B), while dpH_i_/dt in Na^+^‐free HBS was 8.78 ± 2.02% of dpH_i_/dt in Na^+^ containing HBS at pH_i_ 6.68 ± 0.04 (*P *< 0.0001, *n* = 4). This indicates that NHE activity plays the dominant role in HCO_3_^‐^ independent acid extrusion by the CPE at low pH_i_. In other tissues, regulated V‐ATPase activity is dependent on the presence of CO_2_/HCO_3_^‐^ (Pastor‐Soler et al. 2003). In the isolated choroid plexus cells, however, the presence of CO_2_/HCO_3_^‐^ in the perfusate following acidification did not significantly increase the concanamycin‐sensitive, Na^+^‐independent pH_i_ recovery (*n* = 3, ANOVA *P* = 0.95, Fig. 4C). It seems that the Na^+^‐independent pH_i_ recovery is modest compared to NHE activity, especially at low pH_i_ values, and is not augmented by the presence of CO_2_/HCO_3_^‐^.

### Hypocapnia does not lead to redistribution of the V‐ATPase to the plasma membrane in CPECs

**Figure 5:**
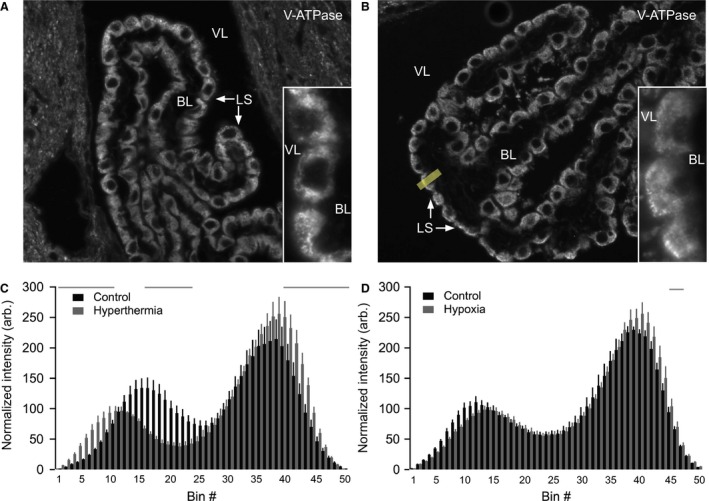
The V‐ATPase does not re‐distribute to the luminal membrane of CPECs in response to hypocapnia. (A) Immunostaining for the V‐ATPase A subunit was performed on brain sections from control mice. (B) Staining was performed in parallel for mice subjected to hyperthermia to induce hypocapnia and thereby respiratory alkalosis. Under these conditions, the V‐ATPase is more closely associated with the apical pole of the epithelial cells. The V‐ATPase staining intensity was quantified by drawing a 21 pixel wide line (yellow line) across each cell with a visible nucleus. The number of basal‐apical bins (measurement points) was then compressed to 50 for each cell. Inserts show high magnification examples of V‐ATPase labeling. (C) Bar graphs showing the average V‐ATPase staining intensity (shown in arbitrary units) for each of the 50 bins in hyperthermia‐treated mice (*n* = 5209 cells) and control mice (*n* = 5227 cells). Bin #1 represents the basal end of the cell, while bin #50 is the luminal end. Error bars represent SEM. Grey horizontal bars represent areas with a significant difference in V‐ATPase staining intensity between control mice and hyperthermia‐treated mice. The quantification shows redistribution of the V‐ATPase toward the apical membrane after hyperthermia‐induced hypocapnia. (D) Similar immunohistochemical analysis of hypoxia‐induced hypocapnia. Bar graphs showing the average V‐ATPase staining intensity (shown in arbitrary units) for each of the 50 bins in hypoxia‐treated mice (*n* = 6296 cells) and control mice (*n* = 6292 cells). Under these conditions, no V‐ATPase redistribution was detectable. CPECs, choroid plexus epithelial cells; VL, ventricle lumen; LS, luminal surface; BL, basolateral interstitium.

In kidney and epididymis, the V‐ATPase is known to react to extra‐ or intracellular pH changes by accumulation in the luminal membrane via activation of sAC (Pastor‐Soler et al. 2003). The possible luminal V‐ATPase membrane accumulation in CPE during alkalization was investigated by decreasing the arterial CO_2_ partial pressure (p_a_CO_2_) in two ways: By increasing the ambient temperature, thereby causing hyperventilation and CO_2_ washout, and by lowering pO_2_ in the ambient air, which similarly leads to hyperventilation. Blood gas analysis confirmed a significant decrease in plasma pCO_2_ and a significant increase in plasma pH after 5 minutes of increased ambient temperature. The p_a_CO_2_ was 4.92 ± 0.25 kPa after increased ambient temperature and 5.98 ± 0.12 kPa under control conditions (*P* = 0.0143, *n* = 13 and 10, respectively), while the pH was 7.41 ± 0.01 after increased ambient temperature and 7.34 ± 0.01 under control conditions (*P* = 0.0017). Immunohistochemical analysis of brain tissue sections from mice subjected to increased ambient temperature showed a statistically significant redistribution of the V‐ATPase toward both the luminal and the basolateral plasma membrane domains of the choroid plexus cells (Fig. 5A–C). Lowering of pO_2_ also produced a significant decrease in plasma pCO_2_ and a significant increase in plasma pH. The pCO_2_ was 5.02 ± 0.31 kPa after lowering of pO_2_ and 6.65 ± 0.10 under control conditions (*P* = 0.0005, *n* = 6), while the pH was 7.37 ± 0.02 after lowering of pO_2_ and 7.30 ± 0.01 under control conditions (*P* = 0.0107). Except for very few bins across the cells, immunohistochemistry revealed statistically insignificant changes in V‐ATPase distribution in the CPE upon lowering pO_2_ (Fig. 5D). Importantly, both protocols apparently failed to induce luminal plasma membrane accumulation of the V‐ATPase in CPE cells.

### Exposure to cAMP increases V‐ATPase activity in CPECs independently of vesicle trafficking

**Figure 6:**
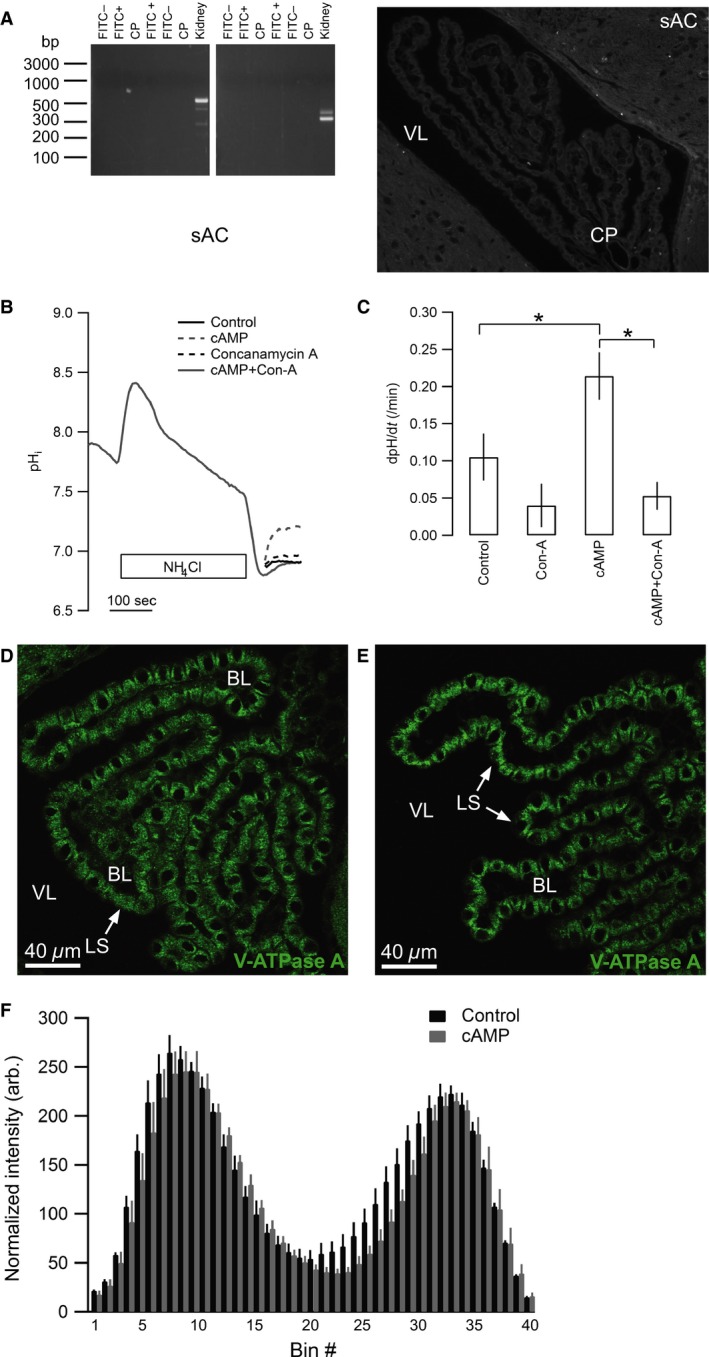
The V‐ATPase in CPECs is stimulated by cAMP independently of membrane trafficking. (A) Expression of sAC in CP was undetectable, analyzed by RT‐PCR on FACS isolated CP cells using two separate primer pairs (left panel). FITC‐ denotes unlabeled CP cells, FITC+ represents isolated surface fluorescein‐lectin labeled cells, CP is the entire choroid plexus. Whole kidney cDNA was used as positive control. Staining for sAC protein immunoreactivity (right panel) was also negative. Low magnification micrograph of the IV ventricle CP. (B) Representative trace of the Na^+^‐independent pH_i_ recovery following acidification by an NH_4_Cl prepulse in isolated clusters of CPE cells from mice. Cells were treated in the recovery period with Na^+^‐free HBS (control), the same solution with 10 mmol/L 8‐CPT cAMP (cAMP), 100 nmol/L concanamycin A, or 10 mmol/L 8‐CPT cAMP and 10 nmol/L concanamycin A (cAMP + Con. A), as indicated. (C) Bar graph showing the mean recovery rates from all experiments (control and cAMP (*n* = 7), concanamycin A and cAMP with concanamycin A (*n* = 4), ANOVA *P* = 0.0037). cAMP stimulated concanamycin‐sensitive pH_i_ recovery in CP cells. Asterisks indicate statistical significance following multiple comparisons posttest. (D) Representative immunohistochemical micrograph of the V‐ATPase localization in mouse CPE after intraventricular installation of control aCSF. (E) Similar staining for the V‐ATPase in mouse CPE after cAMP infusion, showing no apparent redistribution of V‐ATPase staining. (F) Bar graphs showing the average V‐ATPase staining intensity (arbitrary units) for each of the 40 bins in mice after intraventricular injection of cAMP (*n* = 4, 453 cells) or aCSF/control (*n* = 4, 328 cells). Bin #1 represents the basal end, while bin #40 is the luminal end. These data quantitatively confirm the absence of V‐ATPase redistribution after cAMP infusion in vivo. Error bars represent SEM (n.s. at all points). CPE, choroid plexus epithelium; FACS, Fluorescence‐activated cell sorting; VL, ventricle lumen; LS, luminal surface; BL, basolateral interstitium.

In agreement with the lack of membrane accumulation of the V‐ATPase during hypocapnia, mRNA encoding sAC was absent from FACS isolated CPE cells (Fig. 6A), and the CPE failed to display anti‐sAC immunolabeling (Fig. 6B). In kidney proximal tubules and intercalated cells, the V‐ATPase is known to traffic to the apical membrane upon direct stimulation by cAMP, and we therefore speculated that a similar mechanism might operate in CPE cells independently of sAC expression. In isolated clusters of CPE cells, 30 min preincubation in 1 mM 8‐CPT‐cAMP indeed doubled the rate of Na^+^‐independent pH_i_ recovery (*n* = 7, ANOVA *P* = 0.0037, Fig. 6B and C). The increase was completely abolished by concanamycin A, indicating that the increased pH_i_ recovery was caused by V‐ATPase activity. Nevertheless, immunohistochemical analysis of cellular V‐ATPase distribution showed that in vivo cAMP‐injection did not cause statistically significant redistribution of the V‐ATPase in the choroid plexus (*n* = 4, n.s., Fig. 6D, E and F). This indicates a trafficking‐independent activation of the V‐ATPase by cAMP.

## Discussion

In this study, we investigate the expression, activity, and regulation of the V‐ATPase in the choroid plexus epithelium. We show that the choroid plexus expresses the B2 but not the B1 subunit isoform of the V‐ATPase. The V‐ATPase in CPE is mainly found in intracellular compartments both under normal conditions and during alkalinization of the cerebrospinal fluid. Furthermore, the concanamycin A‐sensitive Na^+^‐independent acid extrusion (i.e., V‐ATPase activity) is statistically insignificant in acidified CPE and numerically negligible compared to Na^+^‐dependent acid extrusion (NHE activity) both in the presence and absence of CO_2_/HCO_3_^‐^. The V‐ATPase does not redistribute toward the luminal membrane upon hypocapnia or direct application of cAMP to the brain ventricles. However, membrane‐permeant cAMP increases V‐ATPase activity in the CPE ex vivo.

Research activity in the 1950–1970s revealed the basic mechanisms protecting CSF from major disturbances in acid‐base balance, and the possible involvement of acid/base secretion by the CPE cells in this process (Kazemi and Choma 1977; Vogh and Maren 1975; Husted and Reed 1977). Few studies have been carried out to uncover the molecular mechanisms involved in this regulation. CSF contains very little protein compared to plasma (Boron and Boulpaep 2012) and is, therefore, dependent on the actions of other buffers and/or an appropriate supply of acid /base equivalents to maintain pH. Changes in arterial *p*CO_2_ are directly transferred to the CSF (Siesjoe and Thompson 1965). The resultant CSF pH change, however, is not as large as would be expected by a nonprotein buffered fluid (Johnson et al. 1983). CSF pH is buffered by the CO_2_/HCO_3_^‐^ buffering system, and pH regulation in response to alkaline challenges during hypocapnia is dependent on either removal of HCO_3_^‐^ or transport of H^+^ into the CSF. The choroid plexus is situated in a favorable position for regulating CSF pH and is known to express several integral plasma membrane proteins, which could participate in acid/base transport (Damkier et al. 2013).

We previously demonstrated the expression of the Na^+^/H^+^ exchanger NHE1 (*Slc9a1*) in the luminal CPE membrane (Damkier et al. 2009). This protein transports Na^+^ from the CSF into the cell in exchange for H^+^, indicating a role for this transporter in response to an alkaline CSF. In the current study, we find expression of the A, B2, C1 and d1 subunits of the V‐ATPase in fractions of CPE highly enriched in CP epithelial cells to avoid contamination from the endothelial cells that express many of the same proteins. The protein expression of V‐ATPase subunits in the CPE cells was confirmed by immunoblotting and immunohistochemical staining. This is in agreement with a previous study by Mooradian and Bastani (1993). We mainly observed the immunoreactive proteins in cytosolic compartments, which resemble vesicles by electron microscopy. Nevertheless, the minor fraction of V‐ATPase immunoreactivity observed in the microvilli of the CPE cells is supported by mass spectrometry analysis of luminal surface biotinylated CPE cells, which detected unique peptides for the V‐ATPase subunits a1, a2, A, B2, C1, D, d1, E1, F, G1, G2, H, and the accessory protein S1 (unpublished data).

In several cell types, intracellular V‐ATPase is known to traffic to the plasma membrane to acidify the extracellular fluid (Gluck et al. 1982). This phenomenon has been observed in both renal intercalated cells (Brown et al. 1988b) and in clear cells of the epididymis (Breton et al. 2000). Expression of the B1 subunit has been associated with trafficking to the plasma membrane, as it has been observed in the kidney and epididymis, whereas the ubiquitously expressed B2 subunit is mostly associated with V‐ATPase localization in the lysosomal system. In osteoclasts, however, the B2 subunit is highly expressed in the ruffled membrane, enabling bone matrix degradation (Lee et al. 1996). Furthermore, the B2 subunit has also been detected in the apical membranes of cells in renal proximal tubules and intercalated cells of the collecting duct, suggesting a role in proton secretion and maintenance of acid‐base homeostasis as well (Paunescu et al. 2004, 2007). Thus, the lack of B1 expression in CPE is not indicative of an inability of regulated membrane trafficking of the V‐ATPase complex.

Concanamycin‐sensitive V‐ATPase activity was not detectable in isolated CPE cells at physiological pH_o_. Therefore, to test whether V‐ATPase activity was sensitive to extracellular pH, freshly isolated CP cells were acidified and exposed to varying pH_o_ during the pH_i_ recovery period. We observed a slow Na^+^‐independent pH_i_ recovery that seemed to increase as the pH_o_ increased. This recovery might be attributed to either V‐ATPase activity or to a passive transport of H^+^ out of the cells. However, even after intracellular acidification and pH_i_ recovery at very high extracellular pH (pH 7.8), we find no significant concanamycin A‐sensitive acid extrusion in the CPE cells. This seems to exclude the involvement of V‐ATPase activity in the enhanced acid extrusion. A passive flux of H^+^ would require an appropriate electrochemical driving force under the given experimental conditions. The measured membrane potential (*V*_m_) of approximately −48 mV in freshly isolated mouse CPE cells is close to *V*_m_ values previously observed in rat and mouse CPE cells (personal communication with P.D. Brown). The estimated Veq(H^+^) supports that the driving force for H^+^ is close to zero at pH_o_ 7.0, where no transport is observed, and increases at pH_o_ 7.4 and 7.8, where transport is greatly enhanced. Thus, the observed pH_o_‐sensitive acid extrusion in CPE cells seems better explained by passive H^+^ efflux than by V‐ATPase activity. This efflux could occur through specific membrane proteins, such as yet undescribed H^+^/OH^‐^ transporters, channels, or through leak pathways in the lipid bilayer. Furthermore, the Na^+^‐independent acid extrusion is negligible compared to the acid extrusion in the presence of Na^+^ (NHE1 activity) in CPE cells, suggesting that NHE activity is more likely to be involved in potent acid extrusion by the CPE cells than the probably passive H^+^ efflux.

To investigate whether trafficking of the V‐ATPase in the CPE can be induced by an external stimulus such as an increased CSF pH, we applied two animal models, in which we alkalized the CSF by inducing respiratory alkalosis. Hypocapnia induced by hyperthermia and by hypoxia both caused a decrease in arterial pCO_2_ (p_a_CO_2_). It has previously been shown that the pCO_2_ and pH of CSF rapidly follows changes in p_a_CO_2_, and we confirmed the rise in CSF pH by hypoxia in vivo in the murine system (not shown). In the hypocapnia by hyperthermia model, we find that the V‐ATPase localizes nearer to the luminal membrane compared to nontreated mice but still in subluminal compartments and apparently not in the membrane. In the hypocapnia by hypoxia model, no significant re‐localization took place. This indicates that the intracellular redistribution of V‐ATPase during hyperthermia could be a temperature effect rather than a result of the hypocapnia. In other epithelia, alkalization is sensed by sAC, and the resulting cAMP signal activates PKA‐dependent accumulation of V‐ATPase in the plasma membrane (Gong et al. 2010). However, we do not find evidence for sAC expression in mouse CPE. Thus, the mouse CPE does not seem to contain the HCO_3_^‐^‐sensitive system for V‐ATPase trafficking.

The lack of alkalization‐induced plasma membrane V‐ATPase accumulation did not exclude a direct stimulatory effect of cAMP on V‐ATPase trafficking or function, as observed in epididymis and renal intercalated cells (Paunescu et al. 2010; Pastor‐Soler et al. 2003). Stimulating isolated CPE cells with cAMP induced a concanamycin‐sensitive, Na^+^‐insensitive pH_i_ recovery, that is, V‐ATPase activity. Surprisingly, the apparent induction of V‐ATPase activity ex vivo was not mirrored by V‐ATPase re‐distribution in CPE, when cAMP was injected into the brain ventricles in vivo. One might speculate that cAMP can either directly stimulate the fraction of V‐ATPases already residing in the plasma membrane, or induce intracellular acidification of BCECF‐negative vesicles sufficiently large to reduce cytosolic pH. We have not obtained an animal experiment license to inject concanamycin A into the mouse brain ventricles and are, therefore, prevented from determining the putative effect of cAMP‐induced V‐ATPase activity on CSF pH in vivo. Thus, further studies are necessary to investigate both the short‐term and especially longer‐term effects of cAMP on acid/base transporters including the V‐ATPase in the choroid plexus. At present, we cannot rule out a possible role for the V–ATPase in pH regulation in the CP in longer‐term acid/base disturbances.

In conclusion, the V‐ATPase is expressed mainly in intracellular compartments of CPE cells with minor expression in the luminal plasma membrane. Neither a hypocapnia‐induced increase in CSF pH nor stimulation with cAMP causes accumulation of the V‐ATPase in the luminal plasma membrane of CPE cells. Plasma membrane V‐ATPase activity is negligible both under normal conditions and at high extracellular pH. However, direct application of the membrane‐permeant cAMP analog 8‐CPT‐cAMP to CPE cells stimulates V‐ATPase‐mediated pH_i_ recovery in a trafficking‐independent manner. The transient nature and the magnitude of the cAMP‐stimulated V‐ATPase activity make this mechanism unlikely to mediate physiologically significant contributions to short‐term acidification of the CSF.

## Conflict of Interest

None declared.
